# Illness tracking in SARS-CoV-2 tested persons using a smartphone app: a non-interventional, prospective, cohort study

**DOI:** 10.1016/j.nmni.2022.100967

**Published:** 2022-03-10

**Authors:** T. Lovey, M. Bielecki, N. Gültekin, A. Stettbacher, F. Muggli, Z. Stanga, A. Farnham, J. Deuel, P. Schlagenhauf

**Affiliations:** 1) University of Zürich, Department of Public and Global Health, Epidemiology, Biostatistics and Prevention Institute, Zürich, Switzerland; 2) University Hospital of Zurich, Division of Infectious Diseases and Hospital Hygiene, Switzerland; 3) Swiss Armed Forces, Medical Services, Ittigen, Switzerland; 4) Swiss Armed Forces, Medical Services, Monte Ceneri, Switzerland; 5) University Hospital of Zurich, Division of Medical Oncology and Haematology, Zürich, Switzerland; 6) University of Zürich Centre for Travel Medicine, WHO Collaborating Centre for Travellers' Health, MilMedBiol Competence Centre, Department of Public and Global Health, Epidemiology, Biostatistics and Prevention Institute, Hirschengraben 84, 8001, Zürich, Switzerland

**Keywords:** Ageusia, agnosia, app, COVID-19, illness, omicron, remote monitoring, SARS-CoV-2 infection, symptoms, tiredness

## Abstract

There are few data on the range and severity of symptoms of SARS-CoV-2 infection or the impact on life quality in infected, previously healthy, young adults such as Swiss Armed Forces personnel. It is also unclear if an app can be used to remotely monitor symptoms in persons who test positive. Using a smartphone app called ITITP (Illness Tracking in Tested Persons) and weekly pop-up questionnaires, we aimed to evaluate the spectrum, duration, and impact of symptoms reported after a positive SARS-CoV-2 test according to sex, age, location, and comorbidities, and to compare these to responses from persons who tested negative. We followed up 502 participants (57% active participation), including 68 (13.5%) positive tested persons. Hospitalisation was reported by 6% of the positive tested participants. We found that positives reported significantly more symptoms that are typical of COVID-19 compared to negatives. These symptoms with odds ratio (OR > 1) were *having difficulty breathing* (OR 3.35; 95% CI: 1.16, 9.65; p = 0.03), *having a reduced sense of taste* (OR 5.45; 95% CI: 1.22, 24.34; p = 0.03) and a *reduced sense of smell* (OR 18.24; 95% CI: 4.23, 78.69; p < 0.001). Using a random forest model, we showed that *tiredness* was the single symptom that was rated as having a significant impact on daily activities, whereas the other symptoms, although frequent, had less impact. The study showed that the use of an app was feasible to remotely monitor symptoms in persons infected with SARS-CoV-2 and could be adapted for other settings and new pandemic phases such as the current Omicron wave.

## Introduction

The COVID-19 pandemic, caused by the coronavirus SARS-CoV-2 continues and the epidemiological situation is increasingly complex with new waves of infection and the emergence of highly transmissible virus variants [1]. Vaccines [2], wearing masks [3], social distancing [4], hand hygiene and community mitigation measures [5] are recommended to reduce the impact of the pandemic. There are few data on the range and severity of symptoms or the impact of the infection on life quality in young adults. Data on the persistence of protective immunity after recovery from SARS-CoV-2 infection are scarce [6,7]. Will young people who were infected with an ancestral variant of SARS-CoV-2 produce antibodies that can cross neutralize the emerging and highly transmissible variants such as Omicron, or will they become reinfected with newer variants? Several studies have reported data on patients with severe disease [8] but few articles are evaluating young persons who test positive and who have mild illness or who are asymptomatic. Such data are most important for young population groups as they constitute the workforce of many economies and most likely to engage in social activities and larger events [9]. The Swiss Armed Forces have several bases throughout Switzerland where young recruits and military personnel spend weeks to months as part of their training and military service. During the early period of the pandemic, RT-PCR testing for SARS-CoV-2 was mandatory. We aimed to follow up on those army recruits and personnel with a confirmed positive test for SARS-CoV-2 and compare them to those who tested negative. Our goals were to evaluate the spectrum, duration, severity, and impact of symptoms reported after a positive SARS-CoV-2 test according to sex, age, and location, and compare these to responses from those who tested negative. Furthermore, we aimed to evaluate the feasibility of using an app to identify clusters of symptoms and emerging symptoms such as ageusia (loss of taste functions), anosmia (loss of smell functions) that could be predictive of SARS-CoV-2 infection. We wanted to evaluate symptom duration and impact on daily life activities. A secondary objective was to analyse the geolocation changes of positive cases.

## Methods

To follow up on the spectrum of symptoms, their evolution and resolution we designed a study using a repurposed app for a weekly survey of tested persons (positives, and negatives as a control group) in the army setting. The study app was called ITITP (Illness Tracking in Tested Persons). The University of Zürich Travel Clinic, Epidemiology, Biostatistics, and Prevention Institute (EBPI), and the ETH Wearable Computing Lab partnered in 2014 to develop and field test a smartphone app called “Tourist” for use in travel medicine. Further expertise in using mobile apps to track infection has been gained in a recent project called ITIT (Infection Tracking in Travelers), which received funding in March 2020 from the Swiss National Foundation [10]. We used the knowledge and experience gained in these projects to follow up on army recruits and personnel who tested positive or negative for SARS-CoV-2 before enrolment either with RT-PCR nasopharyngeal tests or serological tests. After approval by the ethical commission of the Republic and Canton of Ticino, Comitato Etico Cantonale (BASEC Number.2020-01146 CE 3637), we recruited participants at Swiss Armed Forces bases in Airolo and Monte Ceneri, Canton Ticino, Switzerland. Inclusion criteria were completion of consent form, being willing and able to participate and to complete the weekly electronic pop-up questionnaires (push notification on smart phone). Tested persons (regardless of whether positive or negative) could participate. After signing an informed consent form, they downloaded the smartphone app using a provided QR code and completed a simple baseline questionnaire simple baseline questions (age, sex, body height, body weight, date of positive test, hospitalised y/n, co-morbidities (such as asthma, high blood pressure), and smoking habits. The pop-up weekly questionnaires were available in English, German, French, and Italian and queried illness symptoms (Appendix 1, BOX 1). All illness symptoms reported were self-rated by the participants using a Likert scale ranging from 1 (none) to 5 (severe). Anyone reporting illness symptoms was asked two additional questions on the impact of illness on their activities and on their general mood (Appendix 1, BOX2). The surveys were anonymous, and the participants could elect to delete their data using a feature on the app.

In addition to the active data received via the weekly survey questionnaires, the app collected passive data only if this was agreed by the participant. Allowing access to passive data was included in the digital consent and was an opt-in or out option. Weekly surveys were geotagged and timestamped when they were filled out and uploaded to the server along with the user ID of the mobile device, which enabled the mapping of locations where the participant completed the survey. The survey upload locations were mapped over time and by whether or not the participant had tested positive or negative.

Data were analysed using R statistical Software Version 4.0.4, R Foundation for statistical computing, Vienna, Austria [11]. Symptom data were evaluated in a random forest model to predict impact on daily activities, and we used the Gini Index to display the impact of the reported symptoms.

## Results

The recruitment started in May 2020 and ended in October 2021.

Socio-demographic characteristics and health determinants.

Of the 502 military personnel who participated in the ITITP project, 14% (68/502) had a positive PCR or a positive SARS-CoV-2 serology test at the time of enrolment. The age of participants ranged from 18 to 60 years, with a mean age of 21 (3.63) years, 12% (58/502) of the participants were female, 27% (122/459) were not in their optimal BMI range: 4.1% (19/459) were classified as underweight and 22.9% (103/459) as overweight, with 6% (19/459) being obese. 35% (178/502) were current smokers, of which 18% (88/502) were daily smokers and 18% (90/502) were occasional smokers. Daily medication use was reported by 7% (34/502) of the soldiers. Comorbidities were reported by 9% (45/502) of participants, distributed as follows: 62% (28/45) asthma, 13% (6/45) hypertension, 7% (3/45) diabetes, 7% (3/45) cancer, 11% (5/45) other diseases (Table 1). Tests for homogeneity between the sociodemographic characteristics and health determinants of our two groups of interest (those who tested positive and those who tested negative) showed no significant differences (Table 1).

**Table 1:** Socio-demographic characteristics and health determinants of the SARS-CoV-2 negative and SARS-CoV-2 positive groups

Characteristic	Overall *N = 502*^a^	SARS-Cov-2 positive (PCR/Serology) *N = 68*^a^	SARS-CoV-2 negative (PCR/Serology) *N = 434*^a^
**Age [years]**	21.55 (3.63) 18.00-60.00	21.06 (1.55) 18.00-25.00	21.63 (3.86) 18.00-60.00
**Gender**			
Female	58 (12%)	7 (10%)	51 (12%)
Male	444 (88%)	61 (90%)	383 (88%)
**BMI [kg/m**^**2**^**]**			
Normal weight	337 (73%)	53 (80%)	284 (72%)
Obese	26 (5.7%)	4 (6.1%)	22 (5.6%)
Overweight	77 (17%)	9 (14%)	68 (17%)
Underweight	19 (4.1%)	0 (0%)	19 (4.8%)
**Smoker**			
Never	310 (62%)	37 (54%)	273 (63%)
Current	88 (18%)	10 (15%)	78 (18%)
Occasional	90 (18%)	17 (25%)	73 (17%)
Former	14 (2.8%)	4 (5.9%)	10 (2.3%)
**Daily medication**	34 (6.8%)	4 (5.9%)	30 (6.9%)
**Comorbidity**	45 (9.0%)	5 (7.4%)	40 (9.2%)

a Mean (SD) Minimum-Maximum; n (%).

### Completion of weekly questionnaires and retention in the study

Of the 502 initial participants, 288 (57%) actively participated in the study by completing at least one questionnaire. A total of 2393 questionnaires were completed, representing an average of five (SD 11) questionnaires per participant. Completion of questionnaires was similar between the sexes (females: eight, SD sixteen; males four, SD ten) (Table 2).

**Table 2:** Comparison of follow-up time and participation in the weekly questionnaire between the SARS-CoV-2 positive and negative groups

Questionnaire *(descriptive analysis)*	Overall *N = 502*^a^	SARS-Cov-2 positive (PCR/Serology) *N = 68*^a^	SARS-CoV-2 negative (PCR/Serology) *N = 434*^a^	*P*-value^b^
**Overall follow-up time [week]**				0.6
< 1 month	142 (29%)	21 (31%)	121 (28%)	
1–3 months	63 (13%)	12 (18%)	51 (12%)	
3–6 months	37 (7.5%)	5 (7.5%)	32 (7.5%)	
6+ months	39 (7.9%)	4 (6.0%)	35 (8.2%)	
No follow-up	214 (43%)	25 (37%)	189 (44%)	
**Number of missed surveys [n]**				0.6
Mean (SD)	3 (5)	3 (5)	3 (6)	
Median (IQR)	0 (0,3)	1 (0,3)	0 (0,3)	
Range	0, 36	0,18	0, 36	
**Survey response rate [%]**				0.3
Mean (SD)	85 (22)	82 (22)	85 (22)	
Median (IQR)	100 (75,100)	86 (70,100)	100 (75,100)	
Range	17, 100	29, 100	17, 100	

a n (%); Mean (SD), Median (IQR), Range.

b Pearson's Chi-squared test; Wilcoxon rank sum test.

Of the 288 participants who completed at least one weekly questionnaire, 49% (140/288) participated for less than one month, 23% (65/288) between one and three months, 14% (39/288) from three to six months, and 14% (39/288) longer than six months (Table 2).

The time between positive PCR or antigen test and response to the first questionnaire (start of follow-up) ranged from zero days to approximately one year with a median of 54 days.

We counted a total of 793 missing questionnaires over the entire observation period, which corresponds to 24% (793/3184) of the missing weekly surveys in our dataset. With a median of eleven weeks for the distribution of total missing weeks, most questionnaires were missed before the third month of follow-up. This corresponds to an average of three (SD five) missing weeks per participant with a maximum of 35 missing questionnaires and a minimum of zero. We can therefore report a survey response rate that varies individually from 17% to 100% and an average of 83% (22) (Table 2).

### Reported symptoms

Having a *runny nose* was the most common symptom, reported by 50% (145/288) of the participants who completed questionnaires during period of the study, followed by *headache* 46% (131/288), and *tiredness* 39% (111/288). Other reported symptoms were a *decreased sense of taste* 10% (29/288) and a *decreased sense of smell* 11.8% (34/288) (Table 3a, Table 3b).

**Table 3a:** Comparison of maximum symptom intensity between the positive and negative SARS-CoV-2 groups

Symptom	N	Overall *N = 502*^1^	SARS-Cov-2 positive (PCR/Serology) *N = 68*^1^	SARS-CoV-2 negative (PCR/Serology) *N = 434*^1^	*P*-value^2^^,^∗
**Cough**	288				ns
None		185 (64%)	27 (63%)	158 (64%)	
Mild		53 (18%)	9 (21%)	44 (18%)	
Moderate		29 (10%)	6 (14%)	23 (9.4%)	
Moderate–-severe		15 (5.2%)	1 (2.3%)	14 (5.7%)	
Severe		6 (2.1%)	0 (0%)	6 (2.4%)	
**Runny nose**	288				ns
None		143 (50%)	20 (47%)	123 (50%)	
Mild		64 (22%)	10 (23%)	54 (22%)	
Moderate		42 (15%)	7 (16%)	35 (14%)	
Moderate–severe		24 (8.3%)	4 (9.3%)	20 (8.2%)	
Severe		15 (5.2%)	2 (4.7%)	13 (5.3%)	
**Sore throat**	288				ns
None		202 (70%)	29 (67%)	173 (71%)	
Mild		42 (15%)	9 (21%)	33 (13%)	
Moderate		24 (8.3%)	2 (4.7%)	22 (9.0%)	
Moderate–severe		13 (4.5%)	2 (4.7%)	11 (4.5%)	
Severe		7 (2.4%)	1 (2.3%)	6 (2.4%)	
**Headache**	288				0.078
None		157 (55%)	16 (37%)	141 (58%)	
Mild		68 (24%)	15 (35%)	53 (22%)	
Moderate		37 (13%)	6 (14%)	31 (13%)	
Moderate–severe		15 (5.2%)	4 (9.3%)	11 (4.5%)	
Severe		11 (3.8%)	2 (4.7%)	9 (3.7%)	
**Difficulty breathing**	288				ns
None		238 (83%)	31 (72%)	207 (84%)	
Mild		31 (11%)	9 (21%)	22 (9.0%)	
Moderate		13 (4.5%)	2 (4.7%)	11 (4.5%)	
Moderate–severe		1 (0.3%)	0 (0%)	1 (0.4%)	
Severe		5 (1.7%)	1 (2.3%)	4 (1.6%)	
**Out of breath**	288				0.053
None		212 (74%)	25 (58%)	187 (76%)	
Mild		46 (16%)	11 (26%)	35 (14%)	
Moderate		19 (6.6%)	6 (14%)	13 (5.3%)	
Moderate–severe		8 (2.8%)	1 (2.3%)	7 (2.9%)	
Severe		3 (1.0%)	0 (0%)	3 (1.2%)	
**Reduced sense of smell**	288				**0.006**
None		254 (88%)	32 (74%)	222 (91%)	
Mild		14 (4.9%)	3 (7.0%)	11 (4.5%)	
Moderate		9 (3.1%)	5 (12%)	4 (1.6%)	
Moderate–severe		8 (2.8%)	2 (4.7%)	6 (2.4%)	
Severe		3 (1.0%)	1 (2.3%)	2 (0.8%)	

1 n (%).

2 Fisher's exact test.

∗ Not significant. Bold values indicates the significant (p-value < = 0.05).

**Table 3b:** Comparison of maximum symptom intensity between the positive and negative SARS-CoV-2 groups

Symptom	N	Overall *N = 502*^1^	SARS-Cov-2 positive (PCR/Serology) *N = 68*^1^	SARS-CoV-2 negative (PCR/Serology) *N = 434*^1^	*P*-value^2^^,^∗
**Reduced sense of taste**	288				**0.031**
None		259 (90%)	35 (81%)	224 (91%)	
Mild		15 (5.2%)	3 (7.0%)	12 (4.9%)	
Moderate		8 (2.8%)	4 (9.3%)	4 (1.6%)	
Moderate–severe		4 (1.4%)	0 (0%)	4 (1.6%)	
Severe		2 (0.7%)	1 (2.3%)	1 (0.4%)	
**Tiredness**	288				ns
None		177 (61%)	25 (58%)	152 (62%)	
Mild		50 (17%)	9 (21%)	41 (17%)	
Moderate		28 (9.7%)	5 (12%)	23 (9.4%)	
Moderate-severe		20 (6.9%)	4 (9.3%)	16 (6.5%)	
Severe		13 (4.5%)	0 (0%)	13 (5.3%)	
**Memory loss**	288				ns
None		219 (76%)	34 (79%)	185 (76%)	
Mild		42 (15%)	6 (14%)	36 (15%)	
Moderate		20 (6.9%)	1 (2.3%)	19 (7.8%)	
Moderate–severe		5 (1.7%)	2 (4.7%)	3 (1.2%)	
Severe		2 (0.7%)	0 (0%)	2 (0.8%)	
**Diarrhoea**	288				ns
None		220 (76%)	35 (81%)	185 (76%)	
Mild		35 (12%)	5 (12%)	30 (12%)	
Moderate		24 (8.3%)	2 (4.7%)	22 (9.0%)	
Moderate–severe		4 (1.4%)	1 (2.3%)	3 (1.2%)	
Severe		5 (1.7%)	0 (0%)	5 (2.0%)	
**Skin symptoms**	288				ns
None		251 (87%)	39 (91%)	212 (87%)	
Mild		21 (7.3%)	1 (2.3%)	20 (8.2%)	
Moderate		11 (3.8%)	2 (4.7%)	9 (3.7%)	
Moderate–severe		2 (0.7%)	0 (0%)	2 (0.8%)	
Severe		3 (1.0%)	1 (2.3%)	2 (0.8%)	
**Night sweating**	288				ns
None		210 (73%)	33 (77%)	177 (72%)	
Mild		38 (13%)	4 (9.3%)	34 (14%)	
Moderate		23 (8.0%)	3 (7.0%)	20 (8.2%)	
Moderate–severe		8 (2.8%)	2 (4.7%)	6 (2.4%)	
Severe		9 (3.1%)	1 (2.3%)	8 (3.3%)	
**Fever**	271				ns
None		214 (79%)	35 (85%)	179 (78%)	
Above 37.5		54 (20%)	6 (15%)	48 (21%)	
Above 39		3 (1.1%)	0 (0%)	3 (1.3%)	

1 n (%).

2 Fisher's exact test.

∗ Not significant.

**Fig. 1:**
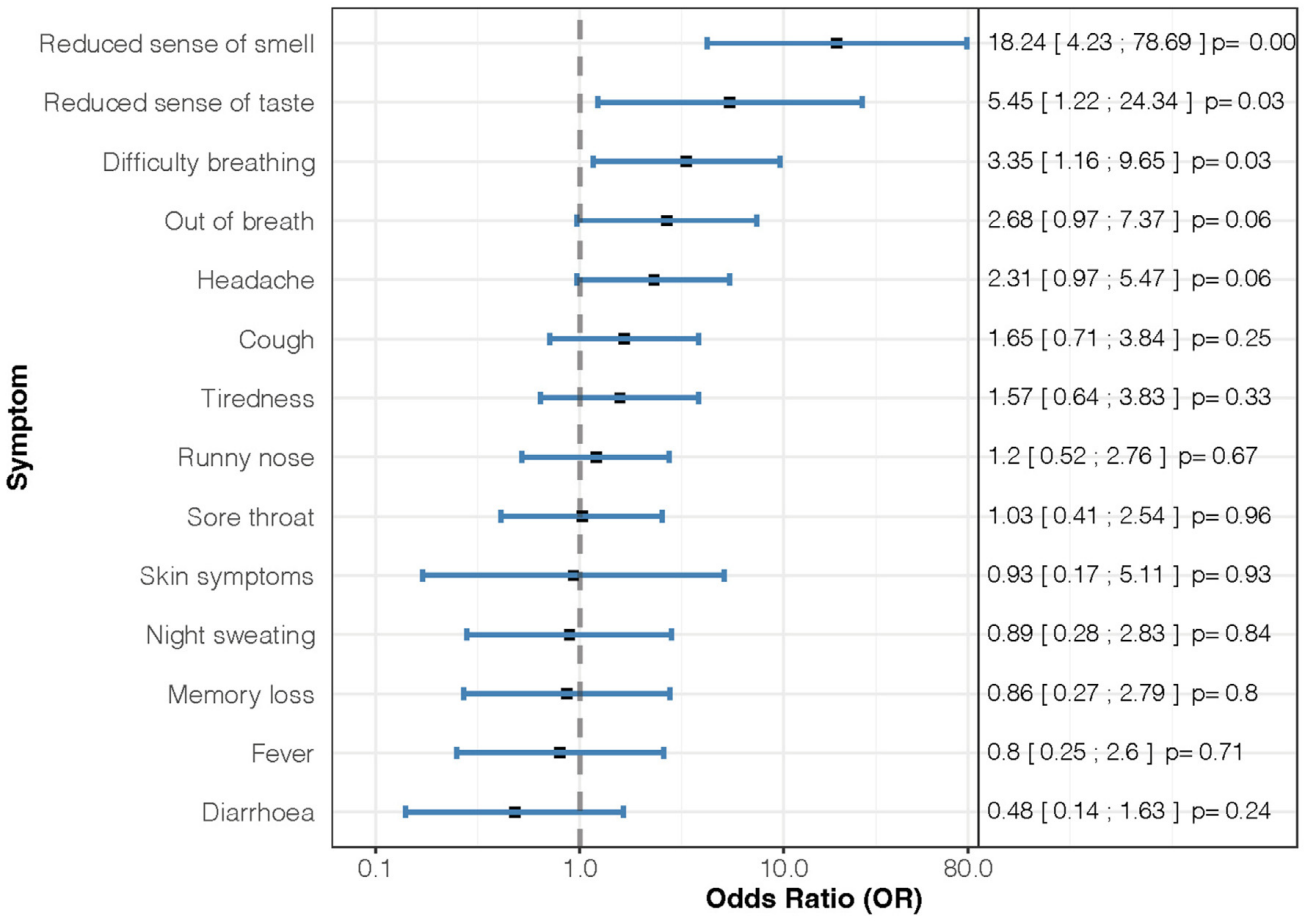
Odds ratio of symptom occurrence in SARS-CoV-2 positive versus SARS-CoV-2 negative person. Odds ratio less than one (symptoms typical of a negative person). Odds ratio greater than one (symptoms typical of a positive person).

Considering the highest score on the Likert scale from *not at all* to *severe*, we found a significant difference between the two groups for *decreased sense of smell* (p = 0.006) and *decreased sense of taste* (p = 0.031). *Headache* (p = 0.078) and *shortness of breath* (p = 0.053) tested just above the significance threshold of alpha 0.05. (Table 3a, Table 3b). Controlling for duration of follow-up and participant individuality, our generalized mixed-effects linear models predict that a participant without infection will express the following symptoms significantly less frequently and with less intensity when baseline demographics remain constant (sex, age, smoking, BMI, medications, comorbidities): *reduced sense of smell* (OR 18.24; 95% CI: 4.23, 78.69; p = 0.00), *reduced sense of taste* (OR 5.45; 95% CI: 1.22, 24.34; p = 0.03) and *difficulty breathing* (OR 3.35; 95% CI: 1.16,9.65; p = 0.03*). Out of breath* (OR 2.68; 95% CI: 0.97, 7.37; p = 0.06) and *headache* (OR 2.31; 95% CI: 0.97,5.47; p = 0.06) also appeared to occur less frequently, but not significantly, in healthy participants (Fig. 1).

### Impact of symptoms on daily activities

**Fig. 2:**
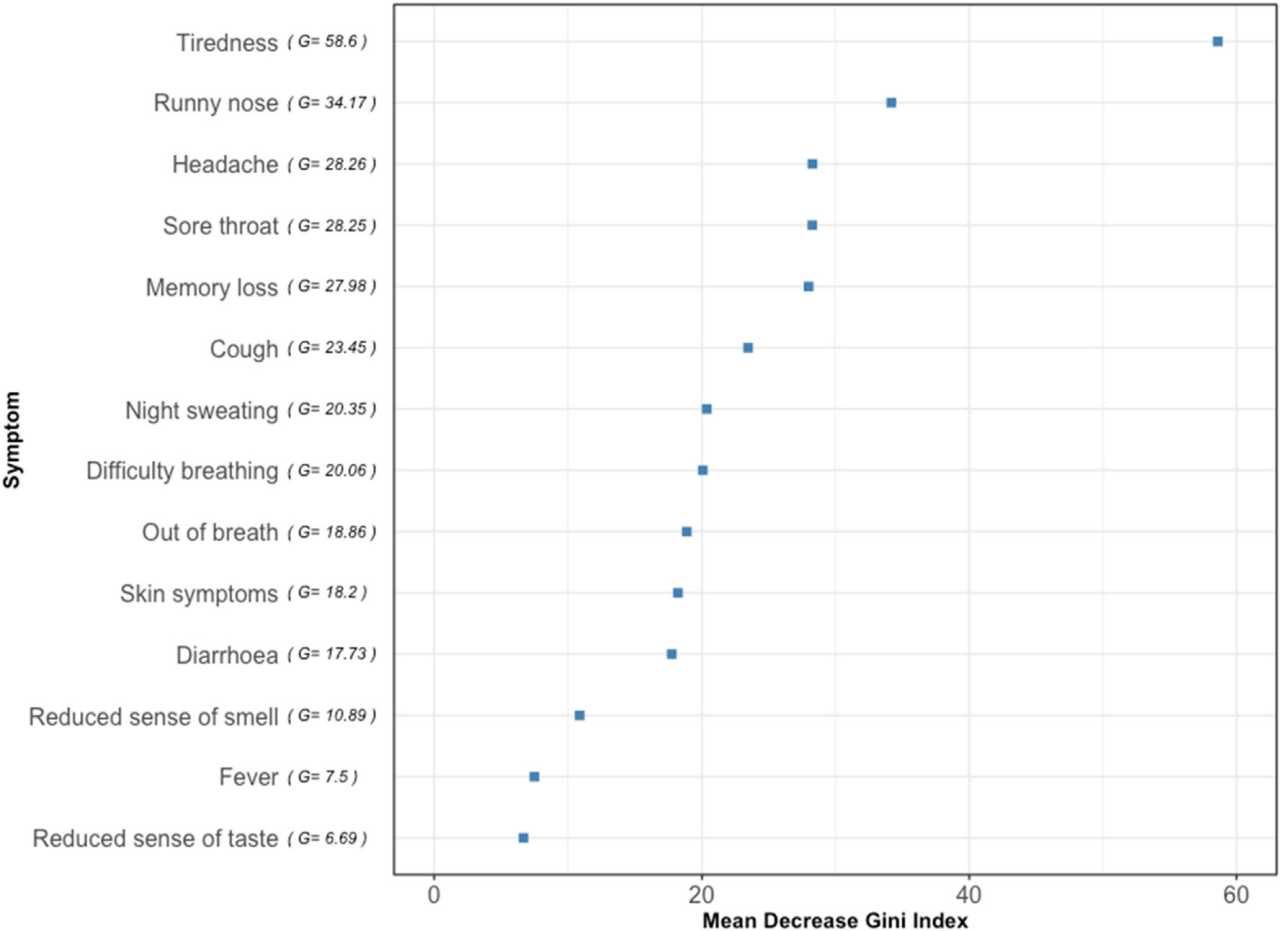
Impact of symptoms on daily activities. The mean decrease in Gini coefficient is a measure of how each variable contributes to the homogeneity of the random forest's nodes and leaves. The greater the mean decrease accuracy or mean decrease Gini score, the greater the importance of the variable in the model and the greater the impact on daily activities (Footer).

**Fig. 3:**
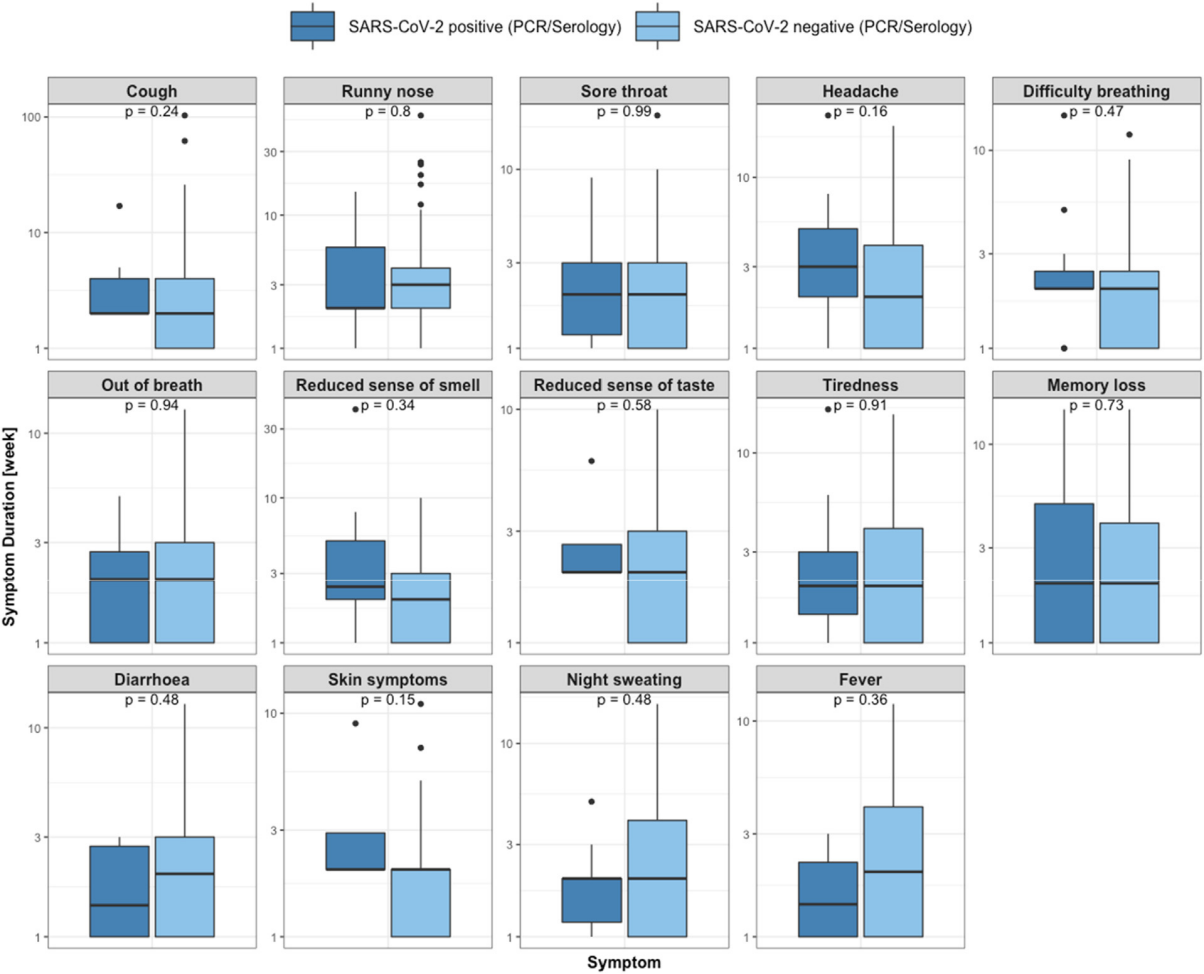
Time in weeks to resolution of symptoms reported by SARS-CoV-2 positive and negative participants.

Using a random forest method, the four different levels of impact (*none*, *mild*, *moderate,* and *severe*) were classified based on the different symptoms that participants experienced during the week. We found that the highest value for the average Gini Index reduction, and thus the most important variable for our model, is *tiredness* (57.19), followed by *runny nose* (34.52). In contrast, some symptoms such as *decreased sense of smell and taste* (10.34, 7.04) or *fever* (7.31) do not seem to affect daily activities (Fig. 2). Symptoms persisted for periods ranging from of mean of 1.38 (SD 1.19) weeks for *diarrhoea* to longer duration symptoms such as *persistent loss of smell* (mean 6 .45, SD 11.44) (Fig. 3).

Four participants (6%, 4/68) reported a severe Covid with hospitalization among the 68 participants who reported a positive PCR result or anti-SRAS-CoV-2 antibody status at enrolment. Nevertheless, no differences were found in their sociodemographic characteristics or health factors compared with the participant with mild COVID-19 illness.

During the study, no reinfections were reported, but one patient reported a first infection after three months of study participation between the second and third waves (12/02/2021). He presented all classic symptoms with high fever (> 39°C), significant impact on his daily activities, described his day as very poor, but did not report hospitalization.

**Fig. 4:**
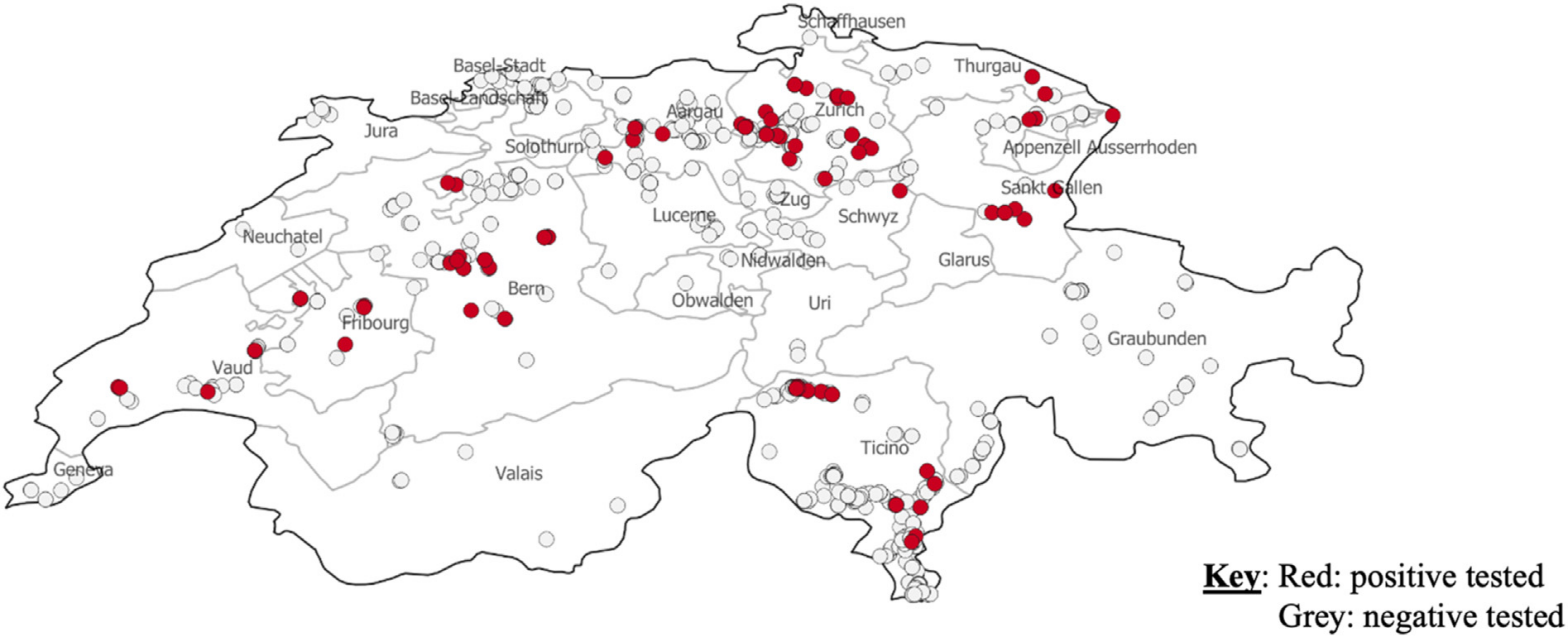
Geolocation of ITITP participants when completing weekly pop-up questionnaires.

Geolocation data did not yield major differences between positives and negatives. Positives in general did not seem to move far with the exception of the one positive that went to Northern Italy (Fig. 4).

## Discussion

Our study showed that use of an app was feasible to remotely monitor symptoms in persons infected with SARS-CoV-2. It was possible to show the range, duration and severity of symptoms experienced, and the impact on daily activities. We showed that those who tested positive for SARS-CoV-2 reported significantly more symptoms that are typical of COVID-19 compared to those who tested negative. These were *having difficulty breathing*, having a *reduced sense of taste (ageusia)* and a *reduced sense of smell (anosmia)*. These results correlate with other studies that describe these typical symptoms in younger populations with COVID-19 [12]. With regard to impact on daily activities, *tiredness* was the single symptom that was rated as a major problem. In contrast, the other symptoms above, although frequent, were not considered to have such a major impact. The finding is corroborated by other articles that describe tiredness, fatigue, and exhaustion in SARS-CoV-2 infected persons [12]. In contrast, a metanalysis describing symptoms of a total of 24,410 adults (mean age 49 years) showed that the cardinal symptoms of COVID-19 are fever and a new persistent cough [13]. In our study, 6% (4/68) of those who tested positive were hospitalized (mean: 22 (SD 0) years, male: 3, female: 1). Another study of young adults aged 18-24 living in the United States recorded a 2.7% hospitalization rate with 0.8% admitted to the ICU [14]. This study shows that symptoms persisted for periods of mean of 1.38 (SD 1.19) weeks for *diarrhoea* to longer duration symptoms such as *persistent loss of smell* mean (6.45 weeks, SD 11.44). This is corroborated by a telephone survey of symptomatic adults (under 35 years old) with mild COVID-19 where 35% did not return to their usual state of health two to three weeks after testing [15].

Several apps focusing on different aspects such as health monitoring, contact tracing, pulse oximeter, thermometer, prevalence, and research apps, emerged during the pandemic [16]. The app, with the largest amount of data and patients recruited, used for symptom tracking is probably the Zoe COVID study app [17,18] which evaluated age- and sex-based discrepancies in early symptoms and also found different sets of relevant features between health-care workers and non-health-care workers. Another app with a similar aim is the CoroNotes app by the University of Tübingen [19] which focuses on the well-being of the users: “Users answer questions on whether they feel well, or whether they are experiencing symptoms like headaches, aching limbs, or a fever, for instance.” Another app, COVID Control App was developed by John Hopkins University for patients to submit their daily body temperature [20]. To the best of our knowledge, ours is the only COVID app study that focuses on a homogenous group of young, previously healthy persons and that combines geolocation with self-reported symptoms over a period of one year. The knowledge gained here could be applied in the future in similar settings to allow for remote monitoring of positive cases in outbreak situations such as in schools, hospitals, companies, or in sport clubs or music groups. The remote surveillance, as pioneered here with the ITITP app, could be further developed so that positive cases could be monitored from their homes but when pre-defined clusters or severe symptoms are reported, an alert could be created that promotes medical intervention.

### Strengths and limitations

Strength of our study was the strong and collaborative response to the call for participants at the Swiss Armed Forces bases in the Canton Ticino. Furthermore, our study population was homogenous, young and familiar with smartphone and app technology, and found the app easy to download and use. Participation in the study was completely voluntary. Retention and the enthusiasm to continue to complete the weekly questionnaires declined over the year of follow-up and this was a limitation.

## Conclusions

Remote monitoring of symptoms in those who test positive for SARS-CoV-2 is feasible. Efforts and incentives are needed to increase retention of volunteers over time. Remote surveillance as in the ITITP study could be further developed for different settings such as schools, sporting clubs, and other organisations, and may be particularly applicable to monitor symptom spectrums in new phases of the pandemic or with the emergence of variants of concern with vaccine escape, such as Omicron.

## Credit author statement

PS designed the study and wrote the protocol. MB, TL, FM, JD, PS were responsible for Data Collection. TL, JD did the analyses, PS, TL, MB, JD drafted the paper. NG, AS, ZS, AF and all authors contributed to the revisions and approved the final version.

## Funding

This work was funded by the Centre of Competence for Military Medicine and Biology at the University of Zürich (MilMedBiol UZH) and the Swiss Army Forces.

## Conflict of interest

None declared.
