# Supplementary material for: Illness tracking in SARS-CoV-2 tested persons using a smartphone app: a non-interventional, prospective, cohort study
Source: New Microbes New Infect. 2022 Mar 10;46:100967. doi: 10.1016/j.nmni.2022.100967 (PMC8908571; doi:10.1016/j.nmni.2022.100967)
Supplement: Multimedia component 1 [file mmc1.docx]

Appendix Box 1. List of questions on the weekly pop-up questionnaire, each rated on a Likert severity scale of 1- 4 (1: mild, 2: moderate, 3: moderate-severe, 4: severe)

| Q1 . Symptoms |
| --- |
| Did you have a cough |
| Did you have fever * |
| Did you have a runny nose |
| Did you have a sore throat |
| Did you have headaches |
| Did you have difficulty breathing |
| Were you easily out of breath |
| Did you have changes in your sense of smell |
| Did you have changes in your sense of taste  Did you feel more tired than usual  Did you have concentration and/or memory problems |
| Did you have diarrhoea  Did you notice any skin reactions such as rash and/or itching  Did you have night-time sweating |
| Did you have other symptoms (please list) |
|  |

*If “yes” then a drop-down menu will ask for more information about the fever. The answer choices are: “not measured” “over 37.5°”, “over 39°”

Appendix Box 2. Drop-down list of questions on the impact of illness symptoms on weekly activities and general mood

| Q2. Impact on activities | | Q3. How would you rate your week overall? |
| --- | --- | --- |
| My illness symptoms did not affect my activities | It was a really bad week | |
| My illness symptoms had a slight negative impact | It was a quite a bad week | |
| My illness symptoms had a moderate negative impact | It was an “OK” week | |
| My illness symptoms had a major negative impact | It was a good week | |
| Due to my symptoms I couldn’t do my daily activities | It was a great week | |
| I had to seek medical attention for my symptoms |  | |
| I tested positive again for SARS-CoV-2 |  | |
| I was hospitalised |  | |
